# Rush Medical College—Opening of the Twenty-third Annual Session—Introductory Address

**Published:** 1866-11

**Authors:** J. W. Freer

**Affiliations:** Professor of Physiology and Surgical Pathology


### CHICAGO MEDICAL JOURNAL.

VOL. XXIII. NOVEMBER, 1866. No. 11.

### ORIGINAL CONTRIBUTIONS.

*Rush Medical College—Opening of the Twenty-Third Annual Session. Introductory Address*, by J. W. FREER, M. D., Professor of Physiology and Surgical Pathology.

GENTLEMEN,—It becomes my pleasant duty, on behalf of my colleagues of the Faculty, to welcome you to these halls of learning.

We need not ask your errand here. You come from your distant homes, the hillsides and valleys of the great West, to reside for a season in the metropolis of the lakes, not for purposes of pleasure, but at a sacrifice of both comfort and means, that you may place yourself in relation with advantages incidental to large cities for the acquirement of medical knowledge.

We hope and believe you will not be disappointed in any of your expectations, for, as heretofore, it will be our constant endeavors to render every facility which ample means under our control, and long experience as teachers of medical science, may enable us to offer.

As an evidence of appreciation, we are pleased to observe so many present on this occasion who have heretofore honored us with their attention.

To those who are as yet strangers among us, we would beg leave to add, that we hope that our mutual labors during the coming session may serve to familiarize us with one another, and that in future you also will give evidence of favor and appreciation by many returns to the institution of your adoption.

Permit me, then, in behalf of the Faculty, to extend to you the hand of fraternal regard, and welcome you to the rewards and labors of the session.

Doubtless you all expect to attain the honorable position of Doctors of Medicine, and to practice the science and art of healing as a calling in life. To secure that object you should become fully apprised of the attainments and qualifications necessary to a respectable position in the profession of your choice.

It has become a trite saying that a candidate for admission to a medical school should have gained what is termed a "good preliminary education." Now this phrase may signify more or less, according to its interpretation by different parties or nationalities.

In Europe, it implies a long and exhaustive university training, to the end that the mind may be stored with much that is useful, and stuffed with more that can be of no practical value—mere lumber and trash, never to be available in future life, or recalled, unless by way of regret that so much valuable time was consumed in the accumulation.

While, in this country, we have, perhaps, erred in the opposite extreme, in not demanding tests of any kind before admission to our medical colleges, the doors being open to all who may choose to enter, each being "a law unto himself," and the sole judge of his fitness for the race. If, perchance, there may have been an over-estimate of qualifications and ability for the contest, the mistake brings forth its legitimate fruits of chagrin and mortification on that final day so much dreaded by all candidates for medical honors.

The custom of throwing the responsibility on the candidate as to fitness to enter upon a course of medical studies may be in accordance with the spirit of our free institutions, in which every man claims the sovereign right to regulate his own conduct, with the privilege of selecting and entering upon any calling, at any time or place, prepared or unprepared. Yet, is this condition of things conducive to the advancement and elevation of the medical profession? Who will not answer negatively? especially if men, as heretofore, are admitted to our ranks whose preparatory education does not embrace even the scanty round of studies taught in a country school-house.

Now, while it might be carrying matters too far to demand what is termed a classical or liberal education, still, would it not be better to require that students before being permitted to enter a medical school should present ample evidence of at least having attained a respectable knowledge of their native language, and whatever else of learning has been acquired, so much the better.

From the generally intelligent appearance of the young gentlemen present, I venture to predict that these modest requirements have been complied with, and more than this, you have doubtless received educations in which the functions of the body, the mind and the heart have been actively exercised and developed, in which the sinews of thought have been strengthened, and habits of attention and concentration acquired. Thus prepared, a student of medicine may enter upon his career with the legitimate hope that his labors in the various sciences will secure a full portion of all that may have been anticipated in moments of the most exalted ambition.

Gentlemen, you enter upon your career as students in stirring times. Progress is stamped upon all human undertakings, and on none more conspicuously than the science of medicine. We are no longer bound over hand and foot to venerable authority and exclusive systems of reasoning. Instead of these we find an independent thought and action prevailing among the great body of scientific workers, evinced by an earnestness of purpose in the evolvement of truth for its own sake and legitimate application, and not as the prop and support of preconceived theories and speculations.

This Faculty profess to be imbued with this spirit of advancement; therefore, your time will not be consumed in listening to cherished hobbies or theories—the deductions from unestablished facts. Your attention will be mainly directed to demonstrable truths—to facts either palpable to the senses, or established by incontrovertible evidence, so that whatever you may hear, see, or observe, may be stored up in your memories as so much real scientific treasure—subject to no discount, and from which aid and comfort may be drawn throughout your professional lives.

The time allotted us is so limited, we can scarcely expect to go beyond the primary elements of the different sciences embraced in the curriculum. Yet these elements are to the abstruser parts what the fundamental rules in arithmetic are to the higher operations in mathematics. It is with these elements that the great problems in medical science are to be wrought. Separated from these, your lives as scientific men will be without foundation or aim.

Teachers of medical science sometimes not only magnify the calling but the importance of their particular branch. Yet all concede to anatomy the first place, without which there can be no scientific medicine or surgery. You will, therefore, cultivate it diligently. It is a subject that cannot be mastered by books and lectures alone, these means being mainly important in giving the language of the science. But, by practicing dissections, by handling, feeling, and seeing, we get a material and practical knowledge that will be retained by the memory long after the language is forgotten. Awful as it is to stand, day after day, in the presence of the dead, the value of dissection is such that it cannot be too strongly insisted upon. It is the very foundation of success, whether for the physician or the surgeon. All those who have risen to eminence, either as physicians or surgeons, have been thorough anatomists. The immortal Harvey revolutionized the entire practice of physic by his knowledge of anatomy. The incessant labors of Hunter in the dissecting room enabled him, aided by the light of genius, to confer such honor upon our profession, and so many benefits upon mankind.

Sir Astley Cooper boasted that he never passed a day, even in the zenith of his practice, without dissecting some part of the body. A physician or surgeon ignorant of anatomy, is like a man professing to mend watches without ever having opened one; or like a traveler wandering in a foreign land, ignorant of its geography, and without chart or compass; or a General at the head of an army, marching and fighting in a country he has never seen—with slender knowledge acquired through danger, clouded by suspicion, and obscured by wilful falsehood.

But the physician can become acquainted with the body, which is the subject of his art, in all its varieties of health and disease, and may thus learn to direct his gaze through skin and fascia, muscle and nerve, down to the very organs of central life. And the surgeon, as he battles for the welfare of his patient in some hazardous operation, may enjoy, as a matter of course, the inestimable advantage of a battle-field which he has already studied expressly as the scene of such a conflict.

It is not for me to point out the details which must demand the attention of the student at different stages of his course. But, when I remind you of its threefold object—that you must dissect to learn the situation or landmarks of the vital organs most concerned in the practice of physic; to recognize the structures, around and within which surgery finds its occupation; and lastly, to gain the operative skill which is only thus to be acquired—you will understand why this practical and personal instruction in anatomy claims so much of your time, and why it comes foremost in the thoughts of a medical teacher, whatever his own special department of instruction. Without it you cannot even begin to acquire a knowledge of many other subjects indispensable to your success as medical scholars. It is the fulcrum of all your subsequent efforts.

You will listen with ever-increasing attention and interest to the lectures on chemistry, with their ample illustrations and beautiful experiments. And when you come to realize the unfolding of the exact laws of this science, with their numerous applications to the useful arts, to the detection of poisons, the nature of disease, and the products of its ravages; of substances for our sustenance, and to the expounding of those physical forces by which we are surrounded, you will feel like lingering on the very threshold of your profession for a more satisfactory survey of this vast storehouse of scientific wealth. At the very beginning of physiological science we are shown its dependence upon chemistry. Without its aid, what would we know of some of the most important functions of the body, of digestion, the nature of secretions and excretions, of the bile, the urine and the gastric juice? We should have no knowledge deserving the name respecting the nature of the blood, the lymph and the textures of the body.

It is almost needless to call your attention to the importance of a knowledge of physiology. It should claim a large share of your attention during your pupilage, and throughout your professional lives. Without this accomplishment you can have no just conception of the laws by which we move and have our being, or of the relations which we sustain to the external world. Anatomy may have been acquired in its broadest sense — descriptive, special and general; chemistry may have yielded her choicest secrets; physics may be as "an oft told tale," and you may have added all other accomplishments of learning, yet, without a special knowledge of physiology, you will lack the very soul and essence of medical science. By it we are permitted to enter the very "Holy of Holies"— to see the workings of the myriad of agents revealed by anatomy, to realize more clearly the nature of that mysterious force about which mankind in every age have been exercised—Life! the motor power of all the living.

A thorough knowledge of its teachings imbues those who practice our calling with an unpretentious spirit — a spirit of modesty and caution while administering to those states termed diseased action, especially when reflecting that neither human skill nor invention has ever yet been able to reproduce the simplest form of life, nor the minutest organic particle in its own proper composition and function, and that all we are permitted to know are some of the conditions necessary to the manifestation of the phenomena of life. The physiological physician does not always see in the materia medica a ready means of regulating the system, of changing the secretions, correcting the liver, and of "putting things to rights" generally, but he often sees the absence of conditions that are necessary to the due performance of the living functions, and supplies them like a good and faithful follower of nature. You will, therefore, pursue with zeal this indispensable branch of medical science, and your reward shall be not only its every day application to the amelioration of the condition of suffering humanity, but your minds will be expanded and ennobled thereby as with no other course of mental training, for you will have had a glimpse with the great poet, and will have seen that,

" Far as creation's ample range extends,
The scale of sensual, mental power ascends.
Mark how it mounts, to man's imperial race,
From the green myriads in the peopled grass;
What modes of sight betwixt each wide extreme,
The mole's dim curtain and the lynx's beam;
Of smell, the headlong lioness between,
And hound sagacious on the tinted green;
Of hearing, from the life that fills the flood,
To that which warbles through the vernal wood.
The spider's touch, how exquisitely fine,
Feels at each thread and lives along the line.
In the nice bee, what senses so subitly true,
From poisonous herb extracts the healing dew.
How instinct varies in the groveling swine,
Compared, half reasoning elephant, with thine.
'Twixt that and reason what a nice barrier;
Forever separate, yet forever near!"

Pathological conditions are the result of irregular physiological action, and are therefore interpreted by the latter. We cannot enter this vast field of perverted life without this biological key. Without this exponent, diseases appear to the minds of men as involving forces and conditions totally distinct from those ordinarily present in the body—as entities—as demons, to be cast out or exorcised with substances of supposed marvelous potency, instead of being simple defects in the nutrition of parts, to be remedied by supplying the conditions of physiological order.

Perceiving, then, the inseparable relation existing between physiological and pathological states—how the former gradually merges into the latter, and so faint is the line of demarkation that it cannot always be distinguished, you will be convinced that without these twin accomplishments you can never interpret disease aright, and, moreover, that your professional lives must necessarily be those of empirics and pretenders, rendering you liable to be seduced into the various *pathies*, *isms* and exclusive systems of the day.

Thus triply armed with anatomical, physiological and pathological learning, you may enter upon the study of the practical branches with profit and pleasure. The teachings of the theory and practice of medicine simply embrace the higher combinations and applications of your attainments in the fundamental sciences.

Disease, to be properly understood, must be read with a physiological eye, the departure from its standard measured by pathology, and the nature of its products be determined by chemistry and morbid anatomy.

The same rules apply to the science of surgery. The physician and surgeon occupy common ground up to the point of operative interference. Here the latter rests upon his anatomical knowledge for the security of his patient and his reputation as a man of skill.

There are those who make bloody and disgraceful records in the world, having no other claims to public confidence than temerity, some mechanical genius, and an indifferent knowledge of anatomy.

But here you will be taught that the greatest triumph in surgical science is that of determining the correct pathology, diagnosis and prognosis of disease, and of judging accurately of the propriety or impropriety of operative procedure.

No true surgeon lops a limb, cleaves a part, or mutilates an organ, without regret that the science has not as yet arrived at that state of perfection to enable him at all times to save rather than destroy.

Thus, every department in medical science is the fruit of a common stem, nourished and developed from a common source, and whatever of difference there may seem betwixt the various specialties in the healing art, to the enlightened mind all are seen to revert back to a common fountain for elucidation.

The science of medicine is said to be progressive; and has it not progressed since the days of Hippocrates, of Galen and of Celsus? Mark the accelerated advancement from the time when the brave and immortal Vesalius, who, contrary to the most violent prejudices of his time, introduced the practice of dissecting the human body. And again, what an impulse was given when the great genius of Harvey discovered the circulation of the blood—an impulse at first scarcely perceptible; but when this great physiological truth had had time to penetrate the thick crust of hallowed errors pertaining to this subject, all the true followers of medical science were electrified with new hope and great prediction of a "good time coming" in the dim future, when medicine would, indeed, be purged of the opprobrium of being but a medley of contradictions, and proudly take her place by the side of the exact sciences. Although to the present time those hopes and predictions have been but partially realized, yet we have reason for increased assurance and encouragement, especially when we come to reflect that, comparatively within a recent period, so many things of vital importance to the advancement of medical science have been placed upon a solid and immutable basis.

With what patience and genius have the great workers in the world of nerve-matter pursued their devious way. They have questioned, racked and tortured the whole scale of animal life for facts and phenomena by which to generalize and develop the laws which govern the mental and physical man. These labors have been rewarded beyond precedent, in placing functions and actions incident to this wonderful system, with its indefinable and inscrutable influence over living tissues, on a basis scarcely less broad than that of the circulation of the life-giving fluids.

What an advancement over former times in the art of diagnosis! With the discoveries of Laennec of auscultation and percussion, a new era dawned upon the medical world. His great heart would have been gladdened could he have been assured that, in less than half a century, optical instruments would be in use capable of revealing in the living subject the deepest recesses of the eye, the intricacies of the larynx, the channel of the urethra, and the cavity of the bladder. Yet, by the genius of man, all these things have been accomplished within the past few years, and by the aid of the laryngoscope and urethroscope, light has been forced around angles and into recesses never before visited by its rays, bringing back the secrets of textural and pathological changes hitherto unrevealed in the living subject.

Doubtless the ancient fathers would have been amazed and startled from their propriety could they have penetrated the future, to behold the surgeons of our time performing the most cruel and formidable operations, with the subjects of their skill unbound and untethered, no sturdy assistants repressing the physical manifestations of an agonizing spirit, but quietly resting in the arms of Lethe until awakened to gladness by the assurance that the terrible ordeal was past.

Did the wildest enthusiasts ever dream that the time would come when the original curse upon woman—"In sorrow shalt thou bring forth children"—should be shorn of its pangs, and the couch of travail be transformed into a downy bed of ease? All these things and more have been accomplished within the memory of the youngest of our number present.

In fact, the improvements, discoveries and amplifications in the different branches have been so extended within recent times that the student finds himself beset with tasks and difficulties of such greater magnitude than those encountered by his predecessors, even of a few years since, that he may well be excused if occasionally found indulging in the weakness of discouragement; and it is simple candor to acknowledge, that there are but few minds capacious enough to contain and make available all that has been attained in the science of medicine and surgery of to-day. Therefore, without by any means wishing to encourage neglect in the acquirement of a thorough knowledge of all the subjects embraced in a course of medical instruction, yet I believe it both proper and expedient to select some department that may be suited to our tastes, genius, or inclinations, on which, in future professional life, to concentrate all of our best energies and attainments. In this way, and in no other, can we become proficient in our calling.

Many now in the full practice of their profession, in looking back through years that have gone, passed in the practical study of disease, remember the feelings of awe with which they contemplated the numerous sciences they had to study, the lectures they were doomed to attend, and the apparent impossibilities which had to be overcome before they could throw aside the title of student and emerge as medical practitioners. For the encouragement of those about to tread the same path, let us add that, by moderate industry and perseverance, all these difficulties will be vanquished. Students may be assured that, with average talent, assiduity and study, they may surmount all that now appears intricate and impracticable. There is no royal road to knowledge, the path is open to all, but it requires constant application to triumph over its obstacles. By working well, ample pleasures and recreations will be won.

In the delights of social intercourse, and in the broad fields of general literature, medical pupils will find inexhaustible sources of gratification. We would especially recommend them to cultivate a taste for reading. Knowledge is a tree whose trunk rises simple and massive, and then parts itself into branches. These branches are laden with fruit, which require but the labor of gathering, a labor which should prove one of love. Whoever has eaten of that fruit in the morning of life, will return to the tree with increased zest in the heat, the burden and the decline of day.

> "Comforts, yea, joys ineffable to find
> Who seek the prouder pleasures of the mind."

Literature and philosophy, sincerely cherished, are friends for life. They multiply and refine our pleasures, soothe our afflictions, soften the pangs of sickness, and embellish and irradiate the most toilsome career.

They must be pursued and cultivated, however, in a proper spirit, always remembering, as Lord Bacon said, the greatest error of all the rest, is the mistaking or misplacing of the last or farthest end of knowledge, for men have entered into a desire of learning and knowledge, sometimes upon a natural curiosity and inquisitive appetite, sometimes to entertain their minds with variety and delight, sometimes for ornament and reputation, and sometimes to enable them to victory of wit and contradiction, and most times for place and profession, and seldom sincerely to give a true account of their gift of reason to the benefit and use of men; as if these sought in knowledge a couch whereon to rest a searching and restless spirit, or a terrace for a wandering and variable mind to walk up and down with a fair prospect, or a tower of state for a proud mind to raise itself upon, or a fort or commanding ground for strife and contention, or a shop for profit and sale, and not a rich storehouse for the glory of the Creator and the relief of man's estate.

